# ColoRobotica: Structured training in robotic colorectal surgery

**DOI:** 10.1111/codi.70247

**Published:** 2025-10-03

**Authors:** Marcos Gómez Ruiz, Samson Tou, Klaus E. Matzel, the ColoRobotica, Shwan Amin, Paolo Bianchi, Peter Coyne, Rogier Crolla, Roland Croner, Nicola Eardley, Eloy Espin‐Basany, Charlie Evans, Havard Forsmo, Carmen Cagigas Fernández, Roger Gerjy, Marcos Gomez Ruiz, Dieter Hahnloser, Jim Khan, Klaus Matzel, Daniel Perez, Lars Thoms Seeberg, Giuseppe Spinoglio, Samson Tou, Matthias Turina, Ellen van Eetvelde, Subash Vasudevan, Marek Zawadzki

**Affiliations:** ^1^ Colorectal Surgery Unit, General Surgery Department Marqués de Valdecilla University Hospital Santander Spain; ^2^ Marqués de Valdecilla Biomedical Research Institute (IDIVAL) Santander Spain; ^3^ Faculty of Medicine University of Cantabria Santander Spain; ^4^ Department of Colorectal Surgery Royal Derby Hospital, University Hospitals of Derby and Burton NHS Foundation Trust Derby UK; ^5^ School of Medicine, Royal Derby Hospital, University of Nottingham Derby UK; ^6^ Department of Surgery Diakoneo Clinic Hallerwiese Nuremberg Germany

**Keywords:** colorectal surgery, ColoRobotica, curriculum, patient outcome, proficiency, robotic surgery, structured training, training

## Abstract

The adoption of robotic surgery has increased rapidly. The robotic surgery market is projected to reach $14 billion globally by 2026, with an increasing number of robotic platforms entering the market. Structured training remains an important issue in robotic colorectal surgery. ColoRobotica at the European School of Coloproctology, the European Society of Coloproctology, was established in 2018 to benchmark robotic colorectal training in Europe. A multidisciplinary team was formed, and a framework was established. Building the infrastructure of the programme took 2 years. A training pathway was designed to provide a structured training programme with quality assurance interventions embedded in the programme. The programme was launched in 2022. Preliminary results showed clinical outcomes of trainees are comparable to those of expert robotic surgeons. The model could serve as a template for both other scientific societies and different specialties to provide structured robotic surgical training.

The adoption of robotic surgery has increased rapidly [1]. The robotic surgery market is projected to reach $14 billion globally by 2026 [2]. In England, the robotic surgical activities have increased from 3099 procedures per year in 2011/12 to 41,134 by 2023/24; a more than 13‐fold increase. In this period, robotic colorectal surgery increased from 178 procedures to 10,160 during the same period [3]. The adoption is indeed in its exponential phase. There are two main challenges facing the adoption of robotic surgeries: (1) cost associated with the purchase of the robotic platforms, consumables and maintenance and (2) structured training.

With the increasing number of robotic platforms entering the market, it is anticipated that competition will drive down the cost of robotic surgical activities. This remains to be seen and is outside the scope of this article. Structured training is crucial to the successful implementation of surgical techniques or new technologies, especially when complex technologies are involved. Lessons have been learned from adverse effects and harms to patients when introducing new techniques, such as in the case of Transanal Total Mesorectal Excision (TaTME) [4]. However, training in robotic surgery has been mainly led by manufacturers with no standardised selection criteria, structured training pathway or assessment for proficiency. Our group set out to provide a framework to benchmark surgical training, specifically robotic colorectal surgery—ColoRobotica [5, 6].

This brief article outlines the process of developing a robotic training programme, its challenges, preliminary clinical and pathological results and future directions. To our knowledge, this training programme is the biggest scientifically structured robotic training programme under the umbrella of a scientific society worldwide.

The Colorectal Robotic Surgery Working Group (CRS WG, ColoRobotica) at the European School of Coloproctology (ESC), European Society of Coloproctology (ESCP), was set up to build a structured training programme and to benchmark robotic colorectal surgery training in 2018. The training programme's goal was to provide a training pathway to equip trainees with proficient knowledge, technical skills and non‐technical skills in robotic colorectal surgery.

It took the group 1 year to secure the grant fund for the training programme, and 2 years to build the infrastructure, which included developing training materials, assessment methodology, a learning management platform and forming a working group (WG) for the training programme. The WG included expert colorectal surgeons with educational interests, educators, anatomists, expert informational technology and graphic design support and a team of administrators. A dedicated database was built in a clinical trial unit (Instituto de Investigación Biomédica Valdecilla (IDIVAL), Santander, Spain).

The ColoRobotica structured training pathway is shown in Figure 1. The stepwise approach aimed to facilitate the safe adoption of robotic colorectal surgery and improve effectiveness, specifically by reducing the learning curve in acquiring robotic colorectal knowledge and skills. There are assessments at every stage of training, and trainees can only proceed to the next stage after passing them. In the clinical phase, trainees are performing initial cases with the presence of our trainers to ensure the safety of the patients and consolidate the structured training. Performance metrics for procedural training were used to allow uniformity in training and provide feedback, as evidence suggests that surgical skills correlate with clinical outcomes [7, 8]. The same metrics were used for assessment when trainees are ready. We are continuously developing and validating these robotic colorectal procedures [9, 10, 11, 12].

**FIGURE 1:**
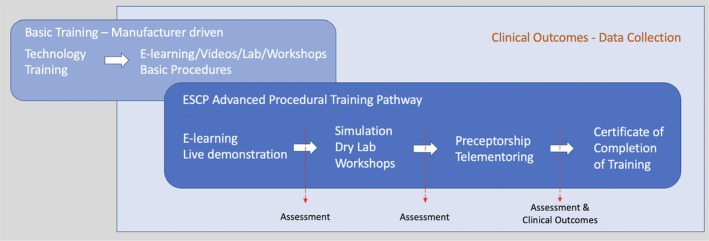
Training pathway.

The final assessment involves the trainees submitting two unedited full colorectal procedural videos (one compulsory procedure is a low anterior rectal resection), which are being assessed blindly by two trainers using the same performance metrics during training. Data, including trainee performance, assessments, clinical outcomes and evaluations, are collected through the learning management platform and REDCap electronic capture tools while trainees go through the training pathway. Trainees are encouraged to submit clinical data after completing their training. Institutional ethics for data collection and General Data Protection Requirements have been obtained from more than 30 participating institutions across geographical Europe. Trainees would be awarded the certification of completion of training upon passing the video assessments and satisfactory clinical outcomes during the training. All our trainers have completed face‐to‐face train‐the‐trainer (TTT) courses specially designed for robotic colorectal surgery to improve training standards and standardise training and assessment methodology [13].

The programme was launched in January 2022. Information about the programme and the selection process criteria was posted on the ESCP platform (Figure 2). Eligible trainees are chosen based on a competitive selection process, including an interview by the programme's Training Programme Committee. One hundred and fifty‐one applicants applied for the programme; 52 (from 14 European countries) were accepted into the programme, of whom seven have not passed or dropped out due to personal reasons.

**FIGURE 2:**
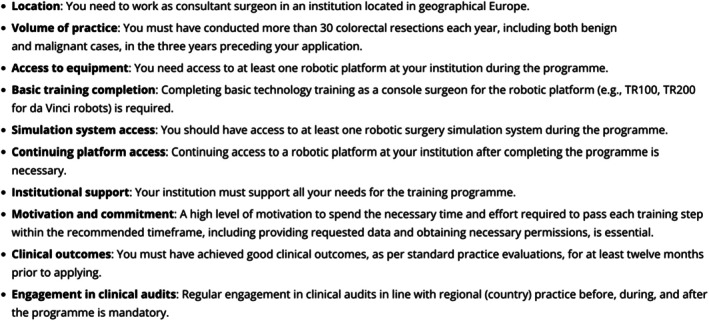
Selection criteria for suitable candidates for the training programme.

We are presenting our preliminary results for the first 334 robotic cases performed by 22 trainees so far, including 76 preceptored cases and 258 solo cases (Table 1). These preliminary results demonstrate good clinical and oncological outcomes, comparable to those published by expert robotic surgeons [14].

**TABLE 1:** Demographics, intraoperative and postoperative data.

	Robotic cases during training programme (*n* = 334)
Age	65.2 (19–92)
Female gender	155 (47.4%)
BMI (kg/m^2^)	27.0 (15.0–50.6)
Indications	
Malignant	252 (75.4%)
Benign	53 (15.9%)
Missing data/to be ascertained	29 (8.7%)
Operations performed (*n* = 326)	
Right hemicolectomy	70 (21.0%)
Left hemicolectomy	26 (7.8%)
Sigmoidectomy	52 (15.6%)
Subtotal colectomy	3 (0.9%)
Total colectomy	1 (0.3%)
High anterior resection	50 (15.0%)
Low anterior resection	83 (24.9%)
Abdomino‐perineal resection of rectum	24 (7.2%)
Rectopexy	17 (5.1%)
Missing data/to be ascertained	8 (2.4%)
Intraoperative parameters	
Operating time, min	271.3 (90–688)
Conversion rate	4 (1.2%)
Estimated blood loss, ml	78.8 (0–1500)
Overall complications	81 (29.8%)
Severe complications (Clavien‐Dindo III or above)	21 (7.7%)
Reoperation	2 (0.6%)
Pathological results (malignant procedures)^a^	
Positive distal resection margin	0 (0%)
Positive circumferential resection margin	3 (1.4%)
Lymph node yield	20.8 (2–52)

^a^ Based on 252 malignant cases.

This endeavour has evolved from traditionally industry‐led to science‐led robotic training. The focus of this programme is on proficiency in surgeons' procedural skills, as surgical skills can significantly impact patient outcomes [15]. The database registry enables feedback on individual surgeons during training and in their early practice, and data analysis would facilitate the correlation of outcomes and metrics along the training pathway.

This short communication documents our journey of setting up a science‐led, structured robotic colorectal training programme in Europe. It is a major endeavour that requires significant expertise, financial resources and time. The premise of setting up this training programme was to benchmark surgical training, in this case, robotic colorectal surgery. To introduce new surgical techniques or procedures, we must ensure the safety of our patients. Steps are taken to ensure the treating teams, not only the operating surgeons, are trained to proficiency before delivering care. While there is a specific learning curve during this period, steps are taken to acquire the necessary knowledge, skills and non‐technical skills before operating on the first patients.

Recommendations have been described to reduce the risks to patients when introducing new techniques or technologies, notably the IDEAL framework [16]. However, resource constraints may render the execution of many initiatives complex. In the ColoRobotica training programme, three additional interventions provide quality‐assured training: (1) The training is a step‐wise progression in obtaining knowledge, skills, non‐technical skills, and each trainee is required to pass the assessments before the next stage of the training pathway, and there is a remedial process if trainees are not meeting the requirements for progression, (2) Each trainer in our programme has attended our TTT course to ensure standardisation of training and assessment methodology. The methodology used is uniform throughout the eLearning materials, face‐to‐face training and assessments. There are regular communications between the Training Programme Committee and the trainers, allowing queries and issues to be clarified and addressed. Some of these interactions led to further improvement of the programme, such as standardising terminology in minimally invasive surgery [13, 17]. (3) Outcomes data during the training pathway, including patients' clinical outcomes. The assessment outcomes during training are not only to determine whether trainees pass the assessments but also to identify areas where trainees need to concentrate more or areas of the course that need improvement (feedback forms are part of the course requirements). The clinical data registry enables the analysis of patient outcome data to monitor adverse outcomes during training. Our team also supports surgeons in contributing to clinical outcomes after they have been signed off from our programme, in line with contributing evidence‐based practice according to the IDEAL framework [9, 16]. The use of performance metrics in our training pathway [10, 11, 12] has the potential to reduce intraoperative errors [7] and shorten the learning curve, thereby improving patient safety and reducing costs.

There are a couple of drawbacks to our programme. Due to the high demand for training and funding, surgeons who fulfil the selection criteria tend to be consultants in their respective countries. The programme has attracted many surgical trainees as demand for robotic training is high [18]. At present, we do not have sufficient resources to fund all applicants participating in our programme, but we do offer alternative training materials, such as webinars [6]. Demand and training are evolving, and we aim to provide high‐standard training to a larger group of trainees soon. Similarly, the programme has attracted an increasing number of surgeons from outside Europe, including those from South America, Asia and Australia. The logistics of some of the face‐to‐face training and funding limit the training outside Europe at present.

Although the programme was designed for robotic colorectal training, some materials developed were useful beyond robotic surgery, such as terminology used in minimally invasive surgery [17], performance metrics in procedural training and assessments, including circular stapling anastomosis [7, 10, 11, 12, 19] and applied anatomy [20].

Although we are in the early phase, the initial results are promising, and our model could serve as a template both for other scientific societies and different specialties to provide structured robotic surgical training.

## AUTHOR CONTRIBUTIONS

**Marcos Gómez Ruiz:** Conceptualization; investigation; funding acquisition; writing – original draft; methodology; validation; writing – review and editing; formal analysis; project administration; data curation; supervision; resources; software. **Samson Tou:** Conceptualization; investigation; funding acquisition; writing – original draft; methodology; validation; writing – review and editing; formal analysis; project administration; data curation; supervision; resources. **Klaus E. Matzel:** Conceptualization; investigation; funding acquisition; writing – original draft; methodology; validation; writing – review and editing; formal analysis; project administration; data curation; supervision; resources.

## FUNDING INFORMATION

The study is part of the ongoing project for ColoRobotica. Intuitive Foundation provided the educational grant for the programme but did not influence the selection of the experts/trainees, the design and conduct of the research, data collection, analysis or preparation of the manuscript.

## CONFLICT OF INTEREST STATEMENT

MGR received grants from Intuitive Surgical, the Intuitive Foundation, and Medtronic. ST received education grants from the Intuitive Foundation and Medtronic. KEM is a Medical Advisor to Medtronic.

## ETHICS STATEMENT

All participants provided informed consent prior to participating in the study. Ethical approval was obtained from the institutional review board.

## Data Availability

The data that support the findings of this study are available from the corresponding author upon reasonable request.

## Working Group of the Colorobotica

Shwan Amin—Sheffield Teaching Hospitals NHS Foundation Trust, Sheffield, UK; Paolo Bianchi—University of Milano, Milano, Italy; Peter Coyne—Newcastle Hospitals NHS Foundation Trust, Newcastle, UK; Rogier Crolla—Amphia Ziekenhuis, Breda, The Netherlands; Roland Croner—Universitat Magdeburg, Magdeburg, Germany; Nicola Eardley—Countess of Chester Hospital NHS Foundation Trust, Chester, UK; Eloy Espin‐Basany—Hospital Valle de Hebron, Barcelona, Spain; Charlie Evans—University Hospitals Coventry and Warwickshire NHS Trust, Coventry, UK; Havard Forsmo—Haukeland University Hospital, Bergen, Norway; Carmen Cagigas Fernández—Hospital Universitario Marqués de Valdecilla, Santander, Spain; Roger Gerjy—Mediclinic Middle East, Dubai, United Arab Emirates; Marcos Gomez Ruiz—Hospital Universitario Marqués de Valdecilla, Santander, Spain; Dieter Hahnloser—Lausanne University Hospital, Lausanne, Switzerland; Jim Khan—Portsmouth Hospitals NHS Trust, Portsmouth, UK; Klaus Matzel—Diakoneo Clinic Hallerwiese, Nuremberg, Germany; Daniel Perez—University Hospital Eppendorf, Hamburg, Germany; Lars Thoms Seeberg—Oslo University Hospital, Oslo, Norway; Giuseppe Spinoglio—Alessandria National Hospital, Alessandria, Italy; Samson Tou—University Hospitals of Derby and Burton NHS Foundation Trust, Derby, UK; Matthias Turina—University of Zurich, Zurich, Switzerland; Ellen van Eetvelde—University Hospital Brussels, Brussels, Belgium; Subash Vasudevan—East Suffolk and North Essex NHS Foundation Trust, Colchester, UK; Marek Zawadzki—Wroclaw University of Science and Technology, Wroclaw, Poland.

## References

[1] Sheetz KH , Claflin J , Dimick JB . Trends in the adoption of robotic surgery for common surgical procedures. JAMA Netw Open. 2020;3:e1918911. 31922557 10.1001/jamanetworkopen.2019.18911 PMC6991252

[2] Positioning the industry for growth in robotic surgery. https://www.oliverwyman.com/our‐expertise/perspectives/health/2023/august/positioning‐the‐industry‐for‐growth‐in‐robotic‐surgery.html. Accessed August 2025

[3] Implementation of robotic‐assisted surgery (RAS) in England. https://gettingitrightfirsttime.co.uk/wp‐content/uploads/2025/07/FINAL_NHS‐England‐and‐GIRFT‐implementation‐of‐robotically‐assisted‐surgery‐in‐England_17‐07‐2025.pdf. Accessed August 2025

[4] Fearnhead NS , Acheson AG , Brown SR , Hancock L , Harikrishnan A , Kelly SB , et al. The ACPGBI recommends pause for reflection on transanal total mesorectal excision. Colorectal Dis. 2020;22:745–748. 32705791 10.1111/codi.15143 PMC7497088

[5] Gomez Ruiz M , Tou S , Matzel KE . Setting a benchmark in surgical training – robotic training under the European School of Coloproctology, ESCP. Colorectal Dis. 2019;21:489–490. 30788899 10.1111/codi.14592

[6] ColoRobotica. https://colorobotica.com/. Accessed August 2025

[7] Mazzone E , Puliatti S , Amato M , Bunting B , Rocco B , Montorsi F , et al. A systematic review and meta‐analysis on the impact of proficiency‐based progression simulation training on performance outcomes. Ann Surg. 2021;274:281–289. 10.1097/SLA.0000000000004650 33630473

[8] Tou S , Gómez Ruiz M , Gallagher AG , Eardley NJ , Matzel KE . Do surgical skills affect outcomes? Colorectal Dis. 2020;22:1826–1829. 10.1111/codi.15316 32790893

[9] Tou S , Au S , Clancy C , Clarke S , Collins D , Dixon F , et al. European Society of Coloproctology guideline on training in robotic colorectal surgery. Colorectal Dis. 2024;26:776–801. 38429251 10.1111/codi.16904 PMC12150670

[10] Tou S , Gómez Ruiz M , Gallagher AG , Matzel KE . European expert consensus on a structured approach to training robotic‐assisted low anterior resection using performance metrics. Colorectal Dis. 2020;22:2232–2242. 32663361 10.1111/codi.15269 PMC7818231

[11] Gómez Ruiz M , Tou S , Gallagher AG , Cagigas Fernández C , Cristobal Poch L , Matzel KE . Intraoperative robotic‐assisted low anterior rectal resection performance assessment using procedure‐specific binary metrics and a global rating scale. BJS Open. 2022;6:zrac041. 10.1093/bjsopen/zrac041 35543264 PMC9092445

[12] Gómez Ruiz M , Bianchi PP , Croner R , Tou S , Matzel K , Gallagher AG , et al. International expert consensus on a structured approach to robotic multiport right hemicolectomy with complete mesocolic excision and intracorporeal anastomosis. Colorectal Dis. 2025;27:e70197. 10.1111/codi.70197 40785273 PMC12336624

[13] Eardley NJ , Matzel KE , Gómez Ruiz M , Khan JS , Riley SA , Donnelly MT , et al. European Society of Coloproctology Colorectal Robotic Surgery Training for the trainers course – the first pilot experience. Colorectal Dis. 2020;22(11):1741–1748. 10.1111/codi.15265 32663345

[14] Egberts JH , Kersebaum JN , Mann B , Aselmann H , Hirschburger M , Graß J , et al. Defining benchmarks for robotic‐assisted low anterior rectum resection in low‐morbid patients: a multicenter analysis. Int J Colorectal Dis. 2021;36:1945–1953. 34244856 10.1007/s00384-021-03988-6 PMC8346389

[15] Birkmeyer JD , Finks JF , O'Reilly A , Oerline M , Carlin AM , Nunn AR , et al. Surgical skill and complication rates after bariatric surgery. N Engl J Med. 2013;369:1434–1442. 10.1056/NEJMsa1300625 24106936

[16] Pennell CP , Hirst AD , Campbell WB , Sood A , Agha RA , Barkun JST , et al. Practical guide to the idea, development and exploration stages of the IDEAL framework and recommendations. Br J Surg. 2016;103:607–615. 10.1002/bjs.10115 26865013

[17] Zawadzki M , Gómez Ruiz M , Tou S , Jeffels A , Matzel KE . A proposed system for standardized terminology in minimally invasive surgery ‐ a video vignette. Colorectal Dis. 2020;22(12):2346–2347. 10.1111/codi.15309 32790087

[18] Fleming CA , Ali O , Clements JM , Hirniak J , King M , Mohan HM , et al. Pan‐specialty access to robotic surgery in surgical training. Br J Surg. 2021;108:e245–e246. 33822004 10.1093/bjs/znab107

[19] Tou S , Gallagher AG , Bislenghi G , Farinha R , Wolthuis A , CAPITAL A Study Collaborative . European expert consensus on a structured approach to circular stapling anastomosis in minimally invasive left‐sided colorectal resection. Colorectal Dis. 2025;27(2):e70037. 10.1111/codi.70037 39980229 PMC11842941

[20] Wedel T , Gómez Ruiz M , Tou S , Stelzner S , Matzel KE . Surgical anatomy of the rectum: a series of video tutorials – a video vignette. Colorectal Dis. 2023;25:1047–1050. 10.1111/codi.16419 36451336

